# (*E*)-1-[2-Hy­droxy-4,6-bis­(meth­oxy­meth­oxy)phen­yl]-3-phenyl­prop-2-en-1-one

**DOI:** 10.1107/S1600536813009380

**Published:** 2013-04-13

**Authors:** Chao Niu, Y. Q. Liu, Y. W. He, H. A. Aisa

**Affiliations:** a Key Laboratory of Plant Resources and Chemistry in Arid Regions, Xinjiang Technical Institute of Physics and Chemistry, University of Chinese Academy of Sciences, People’s Republic of China; b State Key Laboratory Basis of Xinjiang Indigenous Medicinal Plants Resource Utilization, Xinjiang Technical Institute of Physics and Chemistry, Chinese Academy of Sciences, People’s Republic of China

## Abstract

The title compound, C_19_H_20_O_6_, consists of a tetra­substituted benzene ring with one substituent being an α,β-unsaturated cinnamoyl group, which forms an extended conjugated system in the mol­ecule. In addition, two meth­oxy­meth­oxy and one hy­droxy group are bonded to the central benzene ring. The dihedral angle between eh rings is 10.22 (10)°. An intra­molecular hydrogen bond is observed between the hy­droxy group and the carbonyl O atom. One of the meth­oxy­meth­oxy substituents is conformationally disordered over two sets of sites with site-occupation factors of 0.831 (3) and 0.169 (3).

## Related literature

For the preparation of the title compound, see: Sui *et al.* (2012 ▶). For general background to the biological activity of chalcones which posess more than one hy­droxy substituent, see: Jun *et al.* (2007 ▶); Jin *et al.* (2007 ▶); Urgaonkar *et al.* (2005 ▶); Nerya *et al.* (2004 ▶, 2003 ▶); Khatib *et al.* (2005 ▶).
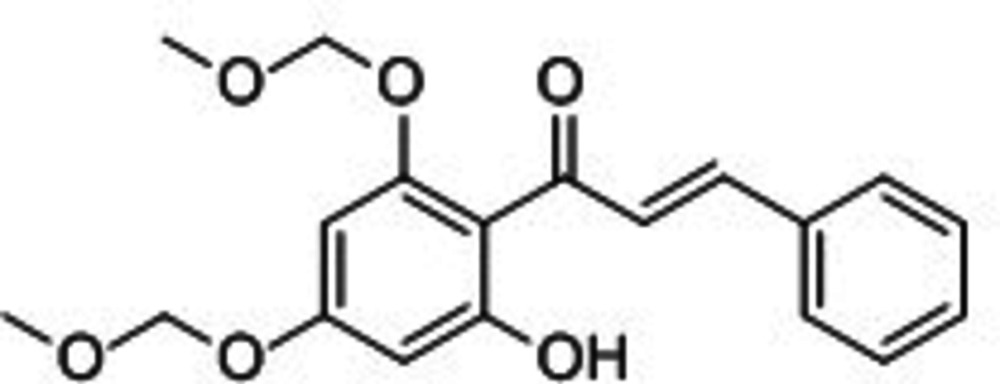


## Experimental

### 

#### Crystal data

C_19_H_20_O_6_
*M* *_r_* = 344.35
Monoclinic, 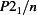

*a* = 8.7791 (2) Å
*b* = 9.7807 (2) Å
*c* = 20.2209 (4) Å
β = 96.792 (2)°
*V* = 1724.10 (6) Å^3^
*Z* = 4
Cu *K*α radiation
μ = 0.82 mm^−1^
*T* = 290 K
0.45 × 0.40 × 0.32 mm

#### Data collection

Agilent Gemini S Ultra diffractometer
Absorption correction: multi-scan (*CrysAlis PRO*; Agilent, 2011) ▶ *T*_min_ = 0.709, *T*_max_ = 0.779
5842 measured reflections
2964 independent reflections
2492 reflections with *I* > 2σ(*I*)
*R*_int_ = 0.045

#### Refinement

*R*[*F* ^2^ > 2σ(*F* ^2^)] = 0.055
*wR*(*F* ^2^) = 0.161
*S* = 1.02
2964 reflections
235 parameters
37 restraints
H-atom parameters constrained
Δρ_max_ = 0.21 e Å^−3^
Δρ_min_ = −0.21 e Å^−3^

### 

Data collection: *CrysAlis PRO* (Agilent, 2011 ▶); cell refinement: *CrysAlis PRO*; data reduction: *CrysAlis PRO*; program(s) used to solve structure: *SHELXS97* (Sheldrick, 2008 ▶); program(s) used to refine structure: *SHELXL97* (Sheldrick, 2008 ▶); molecular graphics: *ORTEP-3 for Windows* (Farrugia, 2012 ▶); software used to prepare material for publication: *SHELXL97*.

## Supplementary Material

Crystal structure: contains datablock(s) I, global. DOI: 10.1107/S1600536813009380/im2424sup1.cif

Structure factors: contains datablock(s) I. DOI: 10.1107/S1600536813009380/im2424Isup2.hkl

Supplementary material file. DOI: 10.1107/S1600536813009380/im2424Isup3.cml

Additional supplementary materials:  crystallographic information; 3D view; checkCIF report

## Figures and Tables

**Table 1:** Hydrogen-bond geometry (Å, °)

*D*—H⋯*A*	*D*—H	H⋯*A*	*D*⋯*A*	*D*—H⋯*A*
O1—H1⋯O2	0.82	1.73	2.466 (2)	148

**Fig. 1.**
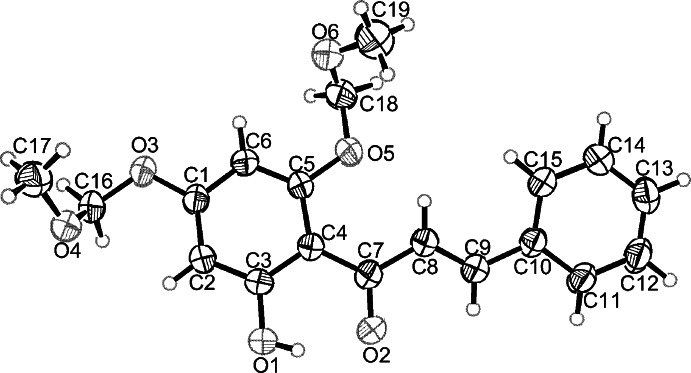
Molecular structure of the title compound with thermal ellipsoids on the 50% probability level.

## supplementary crystallographic information

### Comment

Chalcones are a major class of natural products with widespread distribution in fruits, vegetables, spices, tea and soy based food stuff. They contain two aromatic rings with an unsaturated chain. Many biological activities have been attributed to this group. Recently, the 2',4',6'-trihydroxychalcones were of increasing interest because of their biological properties such as anti-inflammatory activity (Jin *et al.*, 2007), tyrosinase inhibitory activity (Jun *et al.*, 2007) and antidepressant activity (Sui *et al.*, 2012). Many research showed that some chalcones which posess more than one hydroxy substituents on ring A or B act as potential inhibitors of tyrosinase and the position of the hydroxyl group attached to the chalcone rings is of major importance in that activity (Jun *et al.*, 2007; Nerya *et al.*, 2004; Nerya *et al.*, 2003; Khatib *et al.*, 2005).

The structure and the *ORTEP* plot of the molecule are shown in Scheme 1 and Fig. 1. Initially, we assumed that the compound exsisted in only one stable conformation. However, the crystal structure showed two conformational isomers in the crystal structure.

### Experimental

Preparation of the title compound was carried out according to the reported procedure (Sui *et al.*, 2012). Single crystals with sufficient quality for Xray diffraction were prepared by recrystallization from a mixed solution of ethanol and acetone at room temperature.

### Refinement

The C-bound H atoms were placed in ideal positions and were refined as riding on their parent C atoms with C—H = 0.93–0.97 Å and included in the final cycles of refinement using a riding model, with *U*_iso_(H) = 1.2 times *U*_eq_(C). The hydroxy H atom was placed in a calculated position with O—H = 0.82 Å and refined with *U*_iso_(H) = 1.5 times *U*_eq_(O).

### Crystal data

**Table:**

C_19_H_20_O_6_	*F*(000) = 728
*M**_r_* = 344.35	*D*_x_ = 1.327 Mg m^−^^3^
Monoclinic, *P*2_1_/*n*	Melting point: 351 K
Hall symbol: -P 2yn	Cu *K*α radiation, λ = 1.54184 Å
*a* = 8.7791 (2) Å	Cell parameters from 3081 reflections
*b* = 9.7807 (2) Å	θ = 4.4–69.7°
*c* = 20.2209 (4) Å	µ = 0.82 mm^−^^1^
β = 96.792 (2)°	*T* = 290 K
*V* = 1724.10 (6) Å^3^	Block, yellow
*Z* = 4	0.45 × 0.40 × 0.32 mm

### Data collection

**Table:**

Agilent Gemini S Ultra diffractometer	2964 independent reflections
Radiation source: Enhance Ultra (Cu) X-ray Source	2492 reflections with *I* > 2σ(*I*)
Mirror monochromator	*R*_int_ = 0.045
Detector resolution: 15.9149 pixels mm^-1^	θ_max_ = 65.8°, θ_min_ = 4.4°
ω scans	*h* = −10→10
Absorption correction: multi-scan (*CrysAlis PRO*; Agilent, 2011)	*k* = −11→11
*T*_min_ = 0.709, *T*_max_ = 0.779	*l* = −16→23
5842 measured reflections	

### Refinement

**Table:**

Refinement on *F*^2^	Primary atom site location: structure-invariant direct methods
Least-squares matrix: full	Secondary atom site location: difference Fourier map
*R*[*F*^2^ > 2σ(*F*^2^)] = 0.055	Hydrogen site location: inferred from neighbouring sites
*wR*(*F*^2^) = 0.161	H-atom parameters constrained
*S* = 1.02	*w* = 1/[σ^2^(*F*_o_^2^) + (0.0915*P*)^2^ + 0.2682*P*] where *P* = (*F*_o_^2^ + 2*F*_c_^2^)/3
2964 reflections	(Δ/σ)_max_ = 0.001
235 parameters	Δρ_max_ = 0.21 e Å^−^^3^
37 restraints	Δρ_min_ = −0.21 e Å^−^^3^

### Special details

**Table:**

Experimental. CrysAlisPro, Agilent Technologies, Version 1.171.36.21 (release 14-08-2012 CrysAlis171 .NET) (compiled Sep 14 2012,17:21:16) Empirical absorption correction using spherical harmonics, implemented in SCALE3 ABSPACK scaling algorithm.
Geometry. All e.s.d.'s (except the e.s.d. in the dihedral angle between two l.s. planes) are estimated using the full covariance matrix. The cell e.s.d.'s are taken into account individually in the estimation of e.s.d.'s in distances, angles and torsion angles; correlations between e.s.d.'s in cell parameters are only used when they are defined by crystal symmetry. An approximate (isotropic) treatment of cell e.s.d.'s is used for estimating e.s.d.'s involving l.s. planes.
Refinement. Refinement of *F*^2^ against ALL reflections. The weighted *R*-factor *wR* and goodness of fit *S* are based on *F*^2^, conventional *R*-factors *R* are based on *F*, with *F* set to zero for negative *F*^2^. The threshold expression of *F*^2^ > σ(*F*^2^) is used only for calculating *R*-factors(gt) *etc*. and is not relevant to the choice of reflections for refinement. *R*-factors based on *F*^2^ are statistically about twice as large as those based on *F*, and *R*- factors based on ALL data will be even larger.

### Fractional atomic coordinates and isotropic or equivalent isotropic displacement parameters (Å^2^)

**Table:**

	*x*	*y*	*z*	*U*_iso_*/*U*_eq_	Occ. (<1)
O4	−0.21800 (16)	0.09828 (14)	0.66097 (7)	0.0649 (4)	
O1	0.0530 (2)	0.30639 (14)	0.87486 (8)	0.0750 (5)	
H1	0.1167	0.3199	0.9073	0.112*	
O3	−0.19754 (16)	−0.05558 (14)	0.74997 (6)	0.0626 (4)	
O2	0.2224 (2)	0.25370 (14)	0.97783 (8)	0.0742 (5)	
O5	0.12831 (19)	−0.15711 (14)	0.94560 (7)	0.0711 (4)	
C8	0.3002 (2)	0.0383 (2)	1.01680 (9)	0.0574 (5)	
H8	0.3110	−0.0535	1.0063	0.069*	
C2	−0.0697 (2)	0.1321 (2)	0.81172 (9)	0.0563 (5)	
H2	−0.1150	0.1973	0.7822	0.068*	
C7	0.2076 (2)	0.12700 (19)	0.96934 (9)	0.0547 (5)	
C3	0.0291 (2)	0.17122 (18)	0.86711 (9)	0.0541 (5)	
C4	0.10277 (19)	0.07590 (18)	0.91324 (8)	0.0483 (4)	
C6	−0.0300 (2)	−0.10263 (18)	0.84434 (9)	0.0526 (4)	
H6	−0.0489	−0.1948	0.8356	0.063*	
C10	0.4654 (2)	0.00877 (19)	1.12493 (8)	0.0507 (4)	
C9	0.3686 (2)	0.0851 (2)	1.07405 (9)	0.0545 (4)	
H9	0.3533	0.1771	1.0829	0.065*	
C5	0.0665 (2)	−0.06416 (18)	0.89966 (8)	0.0489 (4)	
C15	0.4845 (3)	−0.1320 (2)	1.12293 (10)	0.0659 (5)	
H15	0.4331	−0.1819	1.0880	0.079*	
C13	0.6568 (3)	−0.1276 (3)	1.22351 (11)	0.0702 (6)	
H13	0.7208	−0.1732	1.2562	0.084*	
C14	0.5784 (3)	−0.1987 (2)	1.17194 (12)	0.0745 (6)	
H14	0.5887	−0.2932	1.1700	0.089*	
C1	−0.0993 (2)	−0.00402 (19)	0.80133 (8)	0.0513 (4)	
C16	−0.2928 (2)	0.0378 (2)	0.70981 (10)	0.0615 (5)	
H16A	−0.3823	−0.0104	0.6889	0.074*	
H16B	−0.3278	0.1086	0.7381	0.074*	
C17	−0.1989 (3)	0.0086 (3)	0.60743 (12)	0.0811 (7)	
H17A	−0.1626	0.0593	0.5718	0.122*	
H17B	−0.2955	−0.0332	0.5919	0.122*	
H17C	−0.1257	−0.0610	0.6224	0.122*	
C12	0.6400 (3)	0.0115 (3)	1.22645 (10)	0.0722 (6)	
H12	0.6932	0.0605	1.2612	0.087*	
C11	0.5442 (3)	0.0794 (2)	1.17786 (10)	0.0639 (5)	
H11	0.5326	0.1737	1.1807	0.077*	
C18	0.0761 (3)	−0.2935 (2)	0.94163 (12)	0.0743 (6)	0.831 (3)
H18A	0.1002	−0.3362	0.9849	0.089*	0.831 (3)
H18B	−0.0346	−0.2934	0.9312	0.089*	0.831 (3)
O6	0.1357 (2)	−0.3676 (2)	0.89696 (10)	0.0789 (6)	0.831 (3)
C19	0.2932 (4)	−0.3990 (4)	0.9126 (2)	0.0947 (10)	0.831 (3)
H19A	0.3270	−0.4531	0.8776	0.142*	0.831 (3)
H19B	0.3079	−0.4493	0.9536	0.142*	0.831 (3)
H19C	0.3515	−0.3157	0.9174	0.142*	0.831 (3)
C18'	0.0761 (3)	−0.2935 (2)	0.94163 (12)	0.0743 (6)	0.169 (3)
H18C	−0.0085	−0.3037	0.9680	0.089*	0.169 (3)
H18D	0.0386	−0.3150	0.8958	0.089*	0.169 (3)
O6'	0.1929 (12)	−0.3852 (10)	0.9645 (5)	0.0789 (6)	0.169 (3)
C19'	0.342 (2)	−0.388 (2)	0.9489 (10)	0.0947 (10)	0.169 (3)
H19D	0.3972	−0.4599	0.9731	0.142*	0.169 (3)
H19E	0.3902	−0.3016	0.9608	0.142*	0.169 (3)
H19F	0.3408	−0.4027	0.9019	0.142*	0.169 (3)

### Atomic displacement parameters (Å^2^)

**Table:**

	*U*^11^	*U*^22^	*U*^33^	*U*^12^	*U*^13^	*U*^23^
O4	0.0802 (9)	0.0532 (8)	0.0573 (8)	−0.0038 (6)	−0.0086 (7)	0.0012 (6)
O1	0.0951 (11)	0.0422 (7)	0.0783 (10)	0.0016 (7)	−0.0283 (8)	−0.0011 (6)
O3	0.0701 (8)	0.0561 (8)	0.0555 (7)	−0.0068 (6)	−0.0174 (6)	0.0021 (6)
O2	0.0957 (11)	0.0501 (8)	0.0688 (9)	−0.0009 (7)	−0.0232 (8)	−0.0067 (6)
O5	0.0916 (10)	0.0479 (8)	0.0657 (9)	−0.0043 (7)	−0.0244 (7)	0.0106 (6)
C8	0.0627 (10)	0.0522 (10)	0.0536 (10)	0.0056 (8)	−0.0086 (8)	−0.0059 (8)
C2	0.0620 (11)	0.0489 (10)	0.0540 (10)	0.0024 (8)	−0.0101 (8)	0.0026 (8)
C7	0.0598 (10)	0.0515 (10)	0.0506 (10)	0.0018 (8)	−0.0027 (8)	−0.0065 (8)
C3	0.0606 (10)	0.0419 (9)	0.0573 (10)	0.0025 (8)	−0.0037 (8)	−0.0011 (8)
C4	0.0516 (9)	0.0459 (9)	0.0459 (9)	0.0025 (7)	−0.0010 (7)	−0.0014 (7)
C6	0.0620 (10)	0.0434 (9)	0.0502 (10)	−0.0031 (8)	−0.0029 (8)	0.0007 (7)
C10	0.0521 (9)	0.0538 (10)	0.0449 (9)	−0.0048 (8)	0.0008 (7)	−0.0009 (7)
C9	0.0605 (10)	0.0500 (10)	0.0511 (10)	−0.0027 (8)	−0.0018 (8)	−0.0024 (8)
C5	0.0540 (9)	0.0459 (9)	0.0448 (9)	0.0026 (7)	−0.0023 (7)	0.0032 (7)
C15	0.0747 (13)	0.0573 (12)	0.0600 (11)	−0.0006 (10)	−0.0149 (9)	−0.0074 (9)
C13	0.0664 (12)	0.0832 (15)	0.0569 (11)	0.0044 (11)	−0.0099 (9)	0.0096 (10)
C14	0.0855 (15)	0.0600 (13)	0.0726 (13)	0.0104 (11)	−0.0126 (11)	0.0003 (10)
C1	0.0517 (9)	0.0534 (10)	0.0465 (9)	−0.0024 (8)	−0.0041 (7)	0.0003 (8)
C16	0.0578 (10)	0.0650 (12)	0.0575 (11)	0.0012 (9)	−0.0116 (8)	0.0018 (9)
C17	0.1038 (18)	0.0701 (14)	0.0687 (13)	0.0104 (13)	0.0078 (12)	−0.0033 (11)
C12	0.0782 (13)	0.0797 (15)	0.0529 (11)	−0.0131 (12)	−0.0155 (10)	−0.0021 (10)
C11	0.0804 (13)	0.0566 (11)	0.0510 (10)	−0.0110 (10)	−0.0075 (9)	−0.0017 (8)
C18	0.0887 (14)	0.0545 (11)	0.0760 (13)	−0.0065 (10)	−0.0060 (10)	0.0119 (9)
O6	0.0952 (13)	0.0614 (10)	0.0743 (11)	0.0005 (9)	−0.0151 (9)	0.0004 (8)
C19	0.102 (2)	0.0909 (19)	0.090 (2)	0.0088 (17)	0.0051 (17)	0.0009 (18)
C18'	0.0887 (14)	0.0545 (11)	0.0760 (13)	−0.0065 (10)	−0.0060 (10)	0.0119 (9)
O6'	0.0952 (13)	0.0614 (10)	0.0743 (11)	0.0005 (9)	−0.0151 (9)	0.0004 (8)
C19'	0.102 (2)	0.0909 (19)	0.090 (2)	0.0088 (17)	0.0051 (17)	0.0009 (18)

### Geometric parameters (Å, º)

**Table:**

O4—C16	1.382 (2)	C15—C14	1.376 (3)
O4—C17	1.419 (3)	C15—H15	0.9300
O1—C3	1.345 (2)	C13—C14	1.369 (3)
O1—H1	0.8200	C13—C12	1.370 (3)
O3—C1	1.366 (2)	C13—H13	0.9300
O3—C16	1.426 (2)	C14—H14	0.9300
O2—C7	1.256 (2)	C16—H16A	0.9700
O5—C5	1.366 (2)	C16—H16B	0.9700
O5—C18	1.410 (3)	C17—H17A	0.9600
C8—C9	1.321 (3)	C17—H17B	0.9600
C8—C7	1.466 (3)	C17—H17C	0.9600
C8—H8	0.9300	C12—C11	1.385 (3)
C2—C1	1.368 (3)	C12—H12	0.9300
C2—C3	1.387 (3)	C11—H11	0.9300
C2—H2	0.9300	C18—O6	1.313 (3)
C7—C4	1.462 (2)	C18—H18A	0.9700
C3—C4	1.420 (3)	C18—H18B	0.9700
C4—C5	1.426 (3)	O6—C19	1.415 (4)
C6—C5	1.373 (2)	C19—H19A	0.9600
C6—C1	1.390 (3)	C19—H19B	0.9600
C6—H6	0.9300	C19—H19C	0.9600
C10—C11	1.388 (3)	O6'—C19'	1.38 (2)
C10—C15	1.388 (3)	C19'—H19D	0.9600
C10—C9	1.460 (2)	C19'—H19E	0.9600
C9—H9	0.9300	C19'—H19F	0.9600
			
C16—O4—C17	112.95 (18)	O3—C1—C2	124.58 (16)
C3—O1—H1	109.5	O3—C1—C6	114.20 (17)
C1—O3—C16	118.14 (15)	C2—C1—C6	121.21 (17)
C5—O5—C18	119.41 (16)	O4—C16—O3	112.66 (16)
C9—C8—C7	121.84 (18)	O4—C16—H16A	109.1
C9—C8—H8	119.1	O3—C16—H16A	109.1
C7—C8—H8	119.1	O4—C16—H16B	109.1
C1—C2—C3	118.79 (17)	O3—C16—H16B	109.1
C1—C2—H2	120.6	H16A—C16—H16B	107.8
C3—C2—H2	120.6	O4—C17—H17A	109.5
O2—C7—C4	119.27 (17)	O4—C17—H17B	109.5
O2—C7—C8	117.02 (16)	H17A—C17—H17B	109.5
C4—C7—C8	123.70 (16)	O4—C17—H17C	109.5
O1—C3—C2	116.05 (17)	H17A—C17—H17C	109.5
O1—C3—C4	121.08 (17)	H17B—C17—H17C	109.5
C2—C3—C4	122.86 (17)	C13—C12—C11	120.3 (2)
C3—C4—C5	115.49 (16)	C13—C12—H12	119.8
C3—C4—C7	118.79 (16)	C11—C12—H12	119.8
C5—C4—C7	125.72 (16)	C12—C11—C10	120.9 (2)
C5—C6—C1	120.12 (17)	C12—C11—H11	119.5
C5—C6—H6	119.9	C10—C11—H11	119.5
C1—C6—H6	119.9	O6—C18—O5	114.1 (2)
C11—C10—C15	117.74 (18)	O6—C18—H18A	108.7
C11—C10—C9	118.97 (18)	O5—C18—H18A	108.7
C15—C10—C9	123.28 (17)	O6—C18—H18B	108.7
C8—C9—C10	127.34 (18)	O5—C18—H18B	108.7
C8—C9—H9	116.3	H18A—C18—H18B	107.6
C10—C9—H9	116.3	C18—O6—C19	115.0 (2)
O5—C5—C6	121.93 (16)	O6—C19—H19A	109.5
O5—C5—C4	116.55 (15)	O6—C19—H19B	109.5
C6—C5—C4	121.49 (16)	H19A—C19—H19B	109.5
C14—C15—C10	120.88 (19)	O6—C19—H19C	109.5
C14—C15—H15	119.6	H19A—C19—H19C	109.5
C10—C15—H15	119.6	H19B—C19—H19C	109.5
C14—C13—C12	119.4 (2)	O6'—C19'—H19D	109.5
C14—C13—H13	120.3	O6'—C19'—H19E	109.5
C12—C13—H13	120.3	H19D—C19'—H19E	109.5
C13—C14—C15	120.8 (2)	O6'—C19'—H19F	109.5
C13—C14—H14	119.6	H19D—C19'—H19F	109.5
C15—C14—H14	119.6	H19E—C19'—H19F	109.5
			
C9—C8—C7—O2	15.2 (3)	C3—C4—C5—C6	1.9 (3)
C9—C8—C7—C4	−165.70 (18)	C7—C4—C5—C6	−177.89 (17)
C1—C2—C3—O1	−179.65 (18)	C11—C10—C15—C14	0.1 (3)
C1—C2—C3—C4	0.9 (3)	C9—C10—C15—C14	179.39 (19)
O1—C3—C4—C5	179.44 (18)	C12—C13—C14—C15	0.6 (4)
C2—C3—C4—C5	−1.1 (3)	C10—C15—C14—C13	−0.8 (4)
O1—C3—C4—C7	−0.8 (3)	C16—O3—C1—C2	−9.5 (3)
C2—C3—C4—C7	178.66 (17)	C16—O3—C1—C6	169.82 (16)
O2—C7—C4—C3	4.6 (3)	C3—C2—C1—O3	177.95 (17)
C8—C7—C4—C3	−174.55 (17)	C3—C2—C1—C6	−1.3 (3)
O2—C7—C4—C5	−175.71 (18)	C5—C6—C1—O3	−177.26 (16)
C8—C7—C4—C5	5.2 (3)	C5—C6—C1—C2	2.1 (3)
C7—C8—C9—C10	−178.90 (17)	C17—O4—C16—O3	74.6 (2)
C11—C10—C9—C8	171.4 (2)	C1—O3—C16—O4	82.4 (2)
C15—C10—C9—C8	−7.9 (3)	C14—C13—C12—C11	0.2 (4)
C18—O5—C5—C6	−9.0 (3)	C13—C12—C11—C10	−1.0 (3)
C18—O5—C5—C4	168.81 (18)	C15—C10—C11—C12	0.8 (3)
C1—C6—C5—O5	175.31 (16)	C9—C10—C11—C12	−178.55 (18)
C1—C6—C5—C4	−2.4 (3)	C5—O5—C18—O6	79.7 (3)
C3—C4—C5—O5	−175.96 (16)	O5—C18—O6—C19	70.1 (3)
C7—C4—C5—O5	4.3 (3)		

### Hydrogen-bond geometry (Å, º)

**Table:**

*D*—H···*A*	*D*—H	H···*A*	*D*···*A*	*D*—H···*A*
O1—H1···O2	0.82	1.73	2.466 (2)	148
